# 1,3-Dicyclo­hexyl-1-isonicotinoylurea monohydrate

**DOI:** 10.1107/S1600536808019430

**Published:** 2008-07-09

**Authors:** Cun-Kuan Wang, Feng-Yan Zhou

**Affiliations:** a Faculty of Yang-Ming, Ningbo University, Ningbo, Zhejiang 315211, People’s Republic of China; b Department of Chemistry, Zaozhuang University, Zaozhuang, Shandong 277100, People’s Republic of China

## Abstract

The title organic compound, C_19_H_27_N_3_O_2_·H_2_O, was synthesized from methyl­ene dicyclo­hexyl­amine, 4-pyridine­carboxylic acid and *N*,*N*′-dicyclo­hexyl­carbodiimide. The water molecule is involved in inter­molecular hydrogen bonds, linking symmetry-related urea mol­ecules into a two-dimensional supra­molecular ladder-like structure.

## Related literature

For related literature, see: Iyer *et al.* (1971 ▶); Jew *et al.* (2003 ▶); Li *et al.* (2006 ▶); Mu & Qin (2003 ▶); Wachter *et al.* (1998 ▶).
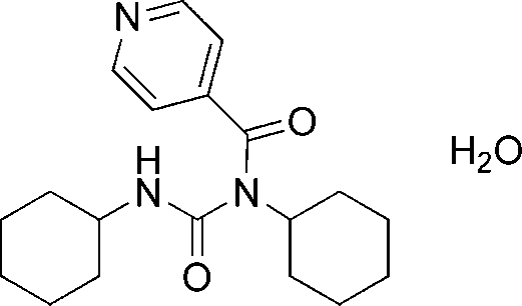


## Experimental

### 

#### Crystal data

C_19_H_27_N_3_O_2_·H_2_O
*M* *_r_* = 347.45
Triclinic, 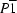

*a* = 6.6694 (13) Å
*b* = 11.106 (2) Å
*c* = 13.248 (3) Å
α = 98.55 (3)°
β = 94.11 (3)°
γ = 97.49 (3)°
*V* = 958.0 (3) Å^3^
*Z* = 2
Mo *K*α radiation
μ = 0.08 mm^−1^
*T* = 298 (2) K
0.40 × 0.33 × 0.28 mm

#### Data collection

Bruker SMART APEXII CCD diffractometer
Absorption correction: multi-scan (*SADABS*; Sheldrick, 1996 ▶) *T*_min_ = 0.968, *T*_max_ = 0.977
9385 measured reflections
4284 independent reflections
2948 reflections with *I* > 2σ(*I*)
*R*_int_ = 0.029

#### Refinement

*R*[*F* ^2^ > 2σ(*F* ^2^)] = 0.047
*wR*(*F* ^2^) = 0.119
*S* = 1.04
4284 reflections
342 parameters
H atoms treated by a mixture of independent and constrained refinement
Δρ_max_ = 0.14 e Å^−3^
Δρ_min_ = −0.19 e Å^−3^

### 

Data collection: *APEX2* (Bruker, 2003 ▶); cell refinement: *SAINT* (Bruker, 2003 ▶); data reduction: *SAINT*; program(s) used to solve structure: *SHELXS97* (Sheldrick, 2008 ▶); program(s) used to refine structure: *SHELXL97* (Sheldrick, 2008 ▶); molecular graphics: *SHELXTL* (Sheldrick, 2008 ▶); software used to prepare material for publication: *SHELXTL*.

## Supplementary Material

Crystal structure: contains datablocks global, I. DOI: 10.1107/S1600536808019430/cs2080sup1.cif

Structure factors: contains datablocks I. DOI: 10.1107/S1600536808019430/cs2080Isup2.hkl

Additional supplementary materials:  crystallographic information; 3D view; checkCIF report

## Figures and Tables

**Table 1:** Hydrogen-bond geometry (Å, °)

*D*—H⋯*A*	*D*—H	H⋯*A*	*D*⋯*A*	*D*—H⋯*A*
O3—H6⋯O1	0.89 (3)	1.95 (3)	2.7959 (18)	158 (2)
N2—H10⋯O3^i^	0.91 (2)	1.89 (2)	2.7949 (19)	170 (2)
O3—H12⋯O2^ii^	0.89 (2)	1.95 (3)	2.8319 (19)	171 (2)

Symmetry codes: (i) 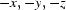; (ii) 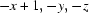.

**Fig. 1.**
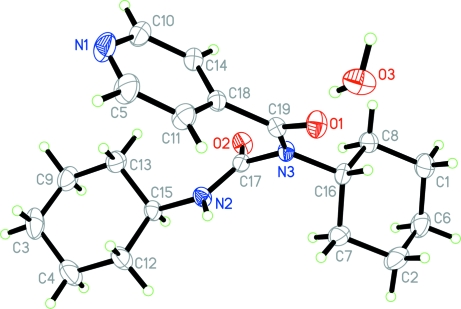
Perspective view of the title complex with the atom-numbering scheme. Atomic displacement ellipsoids are shown at the 30% probability level.

**Fig. 2.**
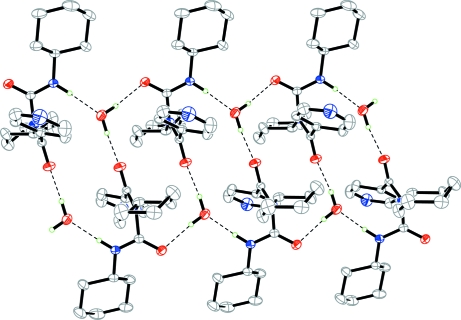
View of the two-dimensional hydrogen-bonded supramolecular structure.

## supplementary crystallographic information

### Comment

Pyridine derivatives are important intermediates widely used in the synthesis of drugs (Wachter *et al.*, 1998; Jew *et al.*, 2003) and pesticides (Li *et al.*, 2006; Mu & Qin, 2003). The title organic compound, 1,3-dicyclohexyl-1-isonicotinoyl-urea, is an intermediate for the synthesis of an anti-tuberculosis drug (Iyer *et al.*, 1971). We report here its synthesis and the crystal structure of its hydrate.

The title compound was synthesized from methylene dicyclohexylamine, 4-pyridinecarboxylic acid and *N*,*N*'-dicyclohexylcarbodiimide. Asymmetric unit of the crystal structure consists of the organic molecule and one H_2_O molecule, C_19_H_27_N_3_O_2_.H_2_O. As shown in Fig. 1 and Table 1, the cyclohexyl groups display chair-type conformation. Interestingly, there are some strong intermolecular hydrogen bonds between the organic molecules and the crystal water. Thus each water effectively links two molecules as O–H···O donor to their O=C groups and accepts one N–H···O hydrogen bridge from a third molecule into a novel two-dimensional supramolecular ladder-like structure through both O—H···O and N—H···O hydrogen bonds (Fig.2 and Table 2).

### Experimental

Methylene dicyclohexylamine (0.21 g, 1 mmol), 4-pyridinecarboxylic acid (0.12 g, 1 mmol) and *N*,*N*'-dicyclohexylcarbodiimide (0.25 g, 1.2 mmol) were added to a 50 ml round bottom flask, then added dichloromethane (25 ml). The mixture was stirred for 12 h at 298 K, after that the reaction mixture was washed with water (10 ml × 3). The organic layer was dried with anhydrous Na_2_SO_4_ and evaporated *in vacuo* to give a residue. The crude product was purified by column chromatography (SiO_2_–EtOAc and hexane, 1:10) to afford the title compound as a colorless solid (yield 69%). ^1^H NMR (400 MHz, CDCl_3_): *d* 8.57 (d, *J* = 5.6 Hz, 2 H), 7.37 (d, *J* = 5.6 Hz, 2 H), 4.16–4.11 (m, 1 H), 3.27–3.22 (m, 1 H), 1.81–1.64 (m, 8 H), 1.60–1.57 (m, 2 H), 1.51–1.22 (m, 4 H), 1.17–1.08 (m, 2 H), 1.00–0.94 (m, 2 H), 0.85–0.76 (m, 2 H).

### Refinement

All H atoms were located in difference Fourier maps and refined independently with isotropic displacement parameters.

### Crystal data

**Table:**

C_19_H_27_N_3_O_2_·H_2_O	*Z* = 2
*M**_r_* = 347.45	*F*_000_ = 376
Triclinic, *P*1	*D*_x_ = 1.205 Mg m^−^^3^
Hall symbol: -P 1	Mo *K*α radiation λ = 0.71073 Å
*a* = 6.6694 (13) Å	Cell parameters from 3059 reflections
*b* = 11.106 (2) Å	θ = 3.1–27.5º
*c* = 13.248 (3) Å	µ = 0.08 mm^−^^1^
α = 98.55 (3)º	*T* = 298 (2) K
β = 94.11 (3)º	Block, colorless
γ = 97.49 (3)º	0.40 × 0.33 × 0.28 mm
*V* = 958.0 (3) Å^3^	

### Data collection

**Table:**

Bruker SMART CCD APEXII diffractometer	4284 independent reflections
Radiation source: fine-focus sealed tube	2948 reflections with *I* > 2σ(*I*)
Monochromator: graphite	*R*_int_ = 0.029
Detector resolution: 8.40 pixels mm^-1^	θ_max_ = 27.5º
*T* = 298(2) K	θ_min_ = 3.1º
ω scans	*h* = −8→7
Absorption correction: multi-scan(SADABS; Sheldrick, 1996)	*k* = −14→14
*T*_min_ = 0.968, *T*_max_ = 0.977	*l* = −17→17
9385 measured reflections	

### Refinement

**Table:**

Refinement on *F*^2^	Secondary atom site location: difference Fourier map
Least-squares matrix: full	Hydrogen site location: inferred from neighbouring sites
*R*[*F*^2^ > 2σ(*F*^2^)] = 0.047	H atoms treated by a mixture of independent and constrained refinement
*wR*(*F*^2^) = 0.119	*w* = 1/[σ^2^(*F*_o_^2^) + (0.054*P*)^2^ + 0.1047*P*] where *P* = (*F*_o_^2^ + 2*F*_c_^2^)/3
*S* = 1.05	(Δ/σ)_max_ < 0.001
4284 reflections	Δρ_max_ = 0.14 e Å^−^^3^
342 parameters	Δρ_min_ = −0.19 e Å^−^^3^
Primary atom site location: structure-invariant direct methods	Extinction correction: none

### Fractional atomic coordinates and isotropic or equivalent isotropic displacement parameters (Å^2^)

**Table:**

	*x*	*y*	*z*	*U*_iso_*/*U*_eq_	
O1	0.25983 (16)	0.03443 (9)	0.01466 (8)	0.0451 (3)	
O2	0.57498 (15)	0.02624 (10)	0.32408 (8)	0.0437 (3)	
O3	0.11137 (18)	−0.00473 (13)	−0.19210 (11)	0.0546 (3)	
N1	0.2456 (3)	−0.40235 (13)	0.05844 (13)	0.0635 (4)	
N2	0.23694 (19)	−0.04403 (11)	0.29989 (9)	0.0357 (3)	
N3	0.38649 (17)	0.06272 (10)	0.18085 (8)	0.0325 (3)	
C1	0.6570 (4)	0.39594 (16)	0.21113 (19)	0.0636 (5)	
C2	0.3121 (3)	0.39686 (17)	0.2711 (2)	0.0667 (6)	
C3	0.0813 (4)	−0.32783 (19)	0.47794 (19)	0.0731 (6)	
C4	−0.0296 (4)	−0.2166 (2)	0.48890 (18)	0.0690 (6)	
C5	0.0909 (3)	−0.34257 (17)	0.03668 (17)	0.0650 (5)	
C6	0.5319 (3)	0.45402 (16)	0.29056 (16)	0.0602 (5)	
C7	0.2898 (3)	0.25718 (15)	0.26796 (16)	0.0536 (5)	
C8	0.6370 (3)	0.25617 (14)	0.20801 (17)	0.0513 (4)	
C9	0.3026 (3)	−0.29165 (18)	0.46441 (15)	0.0593 (5)	
C10	0.4223 (3)	−0.33354 (15)	0.09157 (14)	0.0524 (4)	
C11	0.1057 (3)	−0.21635 (15)	0.04690 (14)	0.0498 (4)	
C12	−0.0014 (3)	−0.1451 (2)	0.40009 (15)	0.0539 (4)	
C13	0.3287 (3)	−0.22376 (17)	0.37377 (14)	0.0505 (4)	
C14	0.4515 (3)	−0.20672 (14)	0.10728 (12)	0.0414 (4)	
C15	0.2219 (2)	−0.11014 (14)	0.38727 (11)	0.0371 (3)	
C16	0.4161 (2)	0.19968 (12)	0.18824 (12)	0.0376 (3)	
C17	0.4089 (2)	0.01254 (12)	0.27488 (10)	0.0326 (3)	
C18	0.2898 (2)	−0.14614 (12)	0.08435 (10)	0.0351 (3)	
C19	0.3093 (2)	−0.00917 (12)	0.09085 (10)	0.0324 (3)	
H1	0.289 (2)	−0.0558 (14)	0.4476 (12)	0.039 (4)*	
H2	0.365 (2)	0.2150 (14)	0.1216 (13)	0.045 (4)*	
H3	0.579 (3)	−0.1618 (15)	0.1334 (12)	0.047 (4)*	
H4	−0.010 (3)	−0.1756 (17)	0.0266 (14)	0.064 (5)*	
H5	0.260 (3)	−0.2770 (17)	0.3092 (15)	0.063 (5)*	
H6	0.153 (4)	−0.014 (2)	−0.128 (2)	0.093 (8)*	
H7	−0.061 (3)	−0.1957 (18)	0.3374 (16)	0.064 (6)*	
H8	0.341 (3)	0.2358 (16)	0.3374 (15)	0.060 (5)*	
H9	0.542 (3)	0.5415 (19)	0.2889 (15)	0.074 (6)*	
H10	0.118 (3)	−0.0381 (17)	0.2633 (15)	0.063 (5)*	
H11	0.686 (3)	0.2370 (19)	0.2787 (17)	0.078 (6)*	
H12	0.212 (4)	−0.019 (2)	−0.2310 (18)	0.084 (7)*	
H13	0.538 (3)	−0.3773 (17)	0.1074 (14)	0.063 (5)*	
H14	0.599 (3)	0.4149 (19)	0.1418 (19)	0.084 (7)*	
H15	0.373 (3)	−0.2369 (19)	0.5286 (17)	0.075 (6)*	
H16	0.706 (3)	0.2202 (18)	0.1530 (16)	0.072 (6)*	
H17	0.473 (3)	−0.1978 (17)	0.3659 (14)	0.065 (6)*	
H18	0.587 (3)	0.4396 (17)	0.3617 (16)	0.067 (6)*	
H19	0.015 (3)	−0.387 (2)	0.4148 (18)	0.086 (7)*	
H20	−0.041 (3)	−0.3951 (19)	0.0133 (16)	0.078 (6)*	
H21	0.258 (3)	0.4170 (19)	0.2025 (18)	0.079 (7)*	
H22	0.150 (3)	0.2190 (17)	0.2552 (14)	0.066 (6)*	
H23	0.067 (3)	−0.369 (2)	0.5385 (18)	0.087 (7)*	
H24	0.018 (4)	−0.160 (2)	0.554 (2)	0.094 (8)*	
H25	0.379 (3)	−0.3669 (19)	0.4574 (15)	0.072 (6)*	
H26	0.235 (3)	0.431 (2)	0.3277 (19)	0.093 (7)*	
H27	−0.064 (3)	−0.0673 (19)	0.4108 (15)	0.071 (6)*	
H28	−0.174 (4)	−0.239 (2)	0.4927 (19)	0.099 (8)*	
H29	0.800 (4)	0.430 (2)	0.2219 (18)	0.091 (7)*	

### Atomic displacement parameters (Å^2^)

**Table:**

	*U*^11^	*U*^22^	*U*^33^	*U*^12^	*U*^13^	*U*^23^
O1	0.0575 (7)	0.0422 (6)	0.0340 (6)	0.0022 (5)	−0.0074 (5)	0.0113 (5)
O2	0.0385 (6)	0.0526 (6)	0.0395 (6)	0.0011 (5)	−0.0045 (5)	0.0143 (5)
O3	0.0408 (6)	0.0825 (9)	0.0413 (7)	0.0172 (6)	−0.0019 (5)	0.0078 (6)
N1	0.0736 (11)	0.0339 (7)	0.0786 (11)	−0.0003 (8)	0.0038 (9)	0.0040 (7)
N2	0.0354 (7)	0.0420 (7)	0.0322 (6)	0.0058 (5)	0.0029 (5)	0.0134 (5)
N3	0.0406 (7)	0.0281 (6)	0.0287 (6)	0.0027 (5)	0.0015 (5)	0.0066 (4)
C1	0.0734 (14)	0.0375 (9)	0.0786 (15)	−0.0047 (9)	0.0201 (11)	0.0097 (9)
C2	0.0732 (14)	0.0413 (10)	0.0859 (16)	0.0170 (10)	0.0152 (12)	−0.0004 (10)
C3	0.1100 (19)	0.0512 (11)	0.0591 (13)	−0.0050 (12)	0.0115 (12)	0.0254 (10)
C4	0.0696 (14)	0.0793 (15)	0.0660 (14)	0.0037 (12)	0.0264 (11)	0.0335 (12)
C5	0.0594 (12)	0.0423 (10)	0.0837 (14)	−0.0081 (9)	−0.0013 (10)	−0.0042 (9)
C6	0.0873 (15)	0.0319 (9)	0.0589 (12)	0.0045 (9)	0.0076 (10)	0.0019 (8)
C7	0.0506 (11)	0.0397 (9)	0.0706 (13)	0.0082 (8)	0.0159 (9)	0.0022 (8)
C8	0.0533 (10)	0.0342 (8)	0.0661 (12)	−0.0008 (7)	0.0210 (9)	0.0059 (8)
C9	0.0894 (15)	0.0476 (10)	0.0478 (11)	0.0204 (10)	0.0105 (10)	0.0192 (8)
C10	0.0619 (11)	0.0392 (9)	0.0578 (11)	0.0116 (9)	0.0042 (9)	0.0102 (8)
C11	0.0461 (9)	0.0397 (9)	0.0580 (11)	−0.0006 (8)	−0.0036 (8)	−0.0007 (7)
C12	0.0492 (10)	0.0645 (12)	0.0537 (11)	0.0056 (9)	0.0152 (9)	0.0253 (9)
C13	0.0656 (12)	0.0488 (9)	0.0435 (10)	0.0182 (9)	0.0106 (9)	0.0172 (8)
C14	0.0468 (9)	0.0357 (8)	0.0411 (8)	0.0023 (7)	0.0018 (7)	0.0082 (6)
C15	0.0449 (8)	0.0391 (8)	0.0289 (7)	0.0050 (7)	0.0041 (6)	0.0108 (6)
C16	0.0530 (9)	0.0272 (7)	0.0323 (8)	0.0048 (6)	0.0011 (6)	0.0064 (6)
C17	0.0387 (8)	0.0289 (7)	0.0302 (7)	0.0056 (6)	0.0026 (6)	0.0049 (5)
C18	0.0427 (8)	0.0320 (7)	0.0291 (7)	0.0006 (6)	0.0047 (6)	0.0039 (5)
C19	0.0321 (7)	0.0336 (7)	0.0309 (7)	0.0013 (6)	0.0026 (6)	0.0067 (6)

### Geometric parameters (Å, °)

**Table:**

O1—C19	1.2253 (16)	C5—H20	0.99 (2)
O2—C17	1.2239 (17)	C6—H9	0.97 (2)
O3—H6	0.89 (3)	C6—H18	1.03 (2)
O3—H12	0.89 (2)	C7—C16	1.516 (2)
N1—C10	1.325 (2)	C7—H8	1.030 (19)
N1—C5	1.335 (3)	C7—H22	0.96 (2)
N2—C17	1.3222 (19)	C8—C16	1.513 (2)
N2—C15	1.4628 (18)	C8—H11	1.03 (2)
N2—H10	0.91 (2)	C8—H16	0.95 (2)
N3—C19	1.3587 (18)	C9—C13	1.518 (2)
N3—C17	1.4443 (17)	C9—H15	1.01 (2)
N3—C16	1.4957 (17)	C9—H25	1.03 (2)
C1—C6	1.511 (3)	C10—C14	1.379 (2)
C1—C8	1.535 (2)	C10—H13	0.988 (19)
C1—H14	1.03 (2)	C11—C18	1.382 (2)
C1—H29	0.97 (2)	C11—H4	0.98 (2)
C2—C6	1.508 (3)	C12—C15	1.518 (2)
C2—C7	1.533 (2)	C12—H7	0.96 (2)
C2—H21	1.02 (2)	C12—H27	1.00 (2)
C2—H26	0.99 (2)	C13—C15	1.522 (2)
C3—C9	1.509 (3)	C13—H5	1.008 (19)
C3—C4	1.515 (3)	C13—H17	0.98 (2)
C3—H19	1.02 (2)	C14—C18	1.382 (2)
C3—H23	0.99 (2)	C14—H3	0.943 (17)
C4—C12	1.523 (3)	C15—H1	0.969 (16)
C4—H24	0.99 (3)	C16—H2	0.970 (17)
C4—H28	0.97 (3)	C18—C19	1.4988 (19)
C5—C11	1.378 (2)		
			
H6—O3—H12	107 (2)	C1—C8—H16	109.9 (12)
C10—N1—C5	116.45 (15)	H11—C8—H16	114.0 (16)
C17—N2—C15	124.07 (13)	C3—C9—C13	111.55 (18)
C17—N2—H10	118.8 (12)	C3—C9—H15	110.0 (12)
C15—N2—H10	117.0 (12)	C13—C9—H15	108.9 (12)
C19—N3—C17	121.70 (11)	C3—C9—H25	111.3 (11)
C19—N3—C16	119.75 (11)	C13—C9—H25	110.8 (11)
C17—N3—C16	117.70 (11)	H15—C9—H25	104.0 (16)
C6—C1—C8	111.14 (16)	N1—C10—C14	124.03 (17)
C6—C1—H14	105.6 (12)	N1—C10—H13	116.8 (11)
C8—C1—H14	109.7 (12)	C14—C10—H13	119.1 (11)
C6—C1—H29	113.1 (14)	C5—C11—C18	118.67 (17)
C8—C1—H29	108.9 (14)	C5—C11—H4	121.7 (11)
H14—C1—H29	108.3 (19)	C18—C11—H4	119.6 (11)
C6—C2—C7	111.16 (17)	C15—C12—C4	111.31 (16)
C6—C2—H21	107.2 (12)	C15—C12—H7	106.9 (12)
C7—C2—H21	110.3 (12)	C4—C12—H7	109.6 (12)
C6—C2—H26	109.6 (13)	C15—C12—H27	107.6 (11)
C7—C2—H26	107.3 (13)	C4—C12—H27	111.5 (11)
H21—C2—H26	111.3 (18)	H7—C12—H27	109.9 (16)
C9—C3—C4	111.04 (17)	C9—C13—C15	110.16 (14)
C9—C3—H19	109.2 (13)	C9—C13—H5	109.9 (11)
C4—C3—H19	107.8 (12)	C15—C13—H5	105.7 (10)
C9—C3—H23	110.5 (13)	C9—C13—H17	111.8 (11)
C4—C3—H23	109.8 (13)	C15—C13—H17	108.5 (11)
H19—C3—H23	108.4 (18)	H5—C13—H17	110.5 (16)
C3—C4—C12	111.67 (18)	C10—C14—C18	118.81 (16)
C3—C4—H24	111.1 (14)	C10—C14—H3	120.8 (10)
C12—C4—H24	108.5 (14)	C18—C14—H3	120.3 (10)
C3—C4—H28	112.3 (14)	N2—C15—C12	108.20 (13)
C12—C4—H28	108.1 (15)	N2—C15—C13	112.09 (12)
H24—C4—H28	105 (2)	C12—C15—C13	110.68 (15)
N1—C5—C11	123.98 (18)	N2—C15—H1	108.0 (9)
N1—C5—H20	115.5 (11)	C12—C15—H1	110.7 (9)
C11—C5—H20	120.5 (12)	C13—C15—H1	107.1 (9)
C2—C6—C1	110.99 (17)	N3—C16—C8	112.88 (13)
C2—C6—H9	109.3 (12)	N3—C16—C7	110.71 (13)
C1—C6—H9	109.7 (12)	C8—C16—C7	111.38 (14)
C2—C6—H18	108.5 (11)	N3—C16—H2	105.2 (9)
C1—C6—H18	108.6 (11)	C8—C16—H2	108.1 (9)
H9—C6—H18	109.8 (16)	C7—C16—H2	108.2 (9)
C16—C7—C2	110.43 (16)	O2—C17—N2	126.20 (13)
C16—C7—H8	107.0 (10)	O2—C17—N3	120.23 (13)
C2—C7—H8	111.0 (10)	N2—C17—N3	113.55 (12)
C16—C7—H22	110.3 (11)	C11—C18—C14	118.01 (14)
C2—C7—H22	112.4 (11)	C11—C18—C19	118.81 (14)
H8—C7—H22	105.5 (15)	C14—C18—C19	123.02 (13)
C16—C8—C1	110.19 (16)	O1—C19—N3	122.14 (13)
C16—C8—H11	105.7 (12)	O1—C19—C18	119.26 (12)
C1—C8—H11	109.8 (12)	N3—C19—C18	118.59 (12)
C16—C8—H16	107.1 (12)		
			
C9—C3—C4—C12	54.2 (3)	C1—C8—C16—N3	178.11 (14)
C10—N1—C5—C11	−0.1 (3)	C1—C8—C16—C7	−56.6 (2)
C7—C2—C6—C1	56.2 (3)	C2—C7—C16—N3	−177.01 (16)
C8—C1—C6—C2	−56.5 (3)	C2—C7—C16—C8	56.5 (2)
C6—C2—C7—C16	−56.0 (3)	C15—N2—C17—O2	−6.0 (2)
C6—C1—C8—C16	56.4 (2)	C15—N2—C17—N3	175.33 (11)
C4—C3—C9—C13	−56.0 (2)	C19—N3—C17—O2	123.81 (15)
C5—N1—C10—C14	2.1 (3)	C16—N3—C17—O2	−66.79 (17)
N1—C5—C11—C18	−1.7 (3)	C19—N3—C17—N2	−57.40 (17)
C3—C4—C12—C15	−54.4 (3)	C16—N3—C17—N2	111.99 (14)
C3—C9—C13—C15	57.5 (2)	C5—C11—C18—C14	1.6 (2)
N1—C10—C14—C18	−2.2 (3)	C5—C11—C18—C19	177.11 (16)
C17—N2—C15—C12	171.48 (14)	C10—C14—C18—C11	0.2 (2)
C17—N2—C15—C13	−66.20 (19)	C10—C14—C18—C19	−175.11 (14)
C4—C12—C15—N2	179.01 (16)	C17—N3—C19—O1	168.24 (12)
C4—C12—C15—C13	55.8 (2)	C16—N3—C19—O1	−0.9 (2)
C9—C13—C15—N2	−178.00 (15)	C17—N3—C19—C18	−13.44 (19)
C9—C13—C15—C12	−57.1 (2)	C16—N3—C19—C18	177.38 (12)
C19—N3—C16—C8	−115.27 (16)	C11—C18—C19—O1	−52.43 (19)
C17—N3—C16—C8	75.12 (17)	C14—C18—C19—O1	122.84 (16)
C19—N3—C16—C7	119.10 (16)	C11—C18—C19—N3	129.19 (15)
C17—N3—C16—C7	−50.51 (18)	C14—C18—C19—N3	−55.53 (19)

### Hydrogen-bond geometry (Å, °)

**Table:**

*D*—H···*A*	*D*—H	H···*A*	*D*···*A*	*D*—H···*A*
O3—H6···O1	0.89 (3)	1.95 (3)	2.7959 (18)	158 (2)
N2—H10···O3^i^	0.91 (2)	1.89 (2)	2.7949 (19)	170 (2)
O3—H12···O2^ii^	0.89 (2)	1.95 (3)	2.8319 (19)	171 (2)

Symmetry codes: (i) −*x*, −*y*, −*z*; (ii) −*x*+1, −*y*, −*z*.
